# Hydrogel Microneedles with Programmed Mesophase Transitions for Controlled Drug Delivery

**DOI:** 10.1021/acsabm.3c01133

**Published:** 2024-02-09

**Authors:** Hala Dawud, Nicole Edelstein-Pardo, Keerthana Mulamukkil, Roey J. Amir, Aiman Abu Ammar

**Affiliations:** † Department of Pharmaceutical Engineering, Azrieli College of Engineering Jerusalem, Jerusalem 9103501, Israel; ‡ School of Chemistry, Faculty of Exact Sciences, Tel-Aviv University, Tel-Aviv 6997801, Israel; § The Center for Physics and Chemistry of Living Systems, Tel-Aviv University, Tel-Aviv 6997801, Israel; ∥ The Center for Nanoscience and Nanotechnology, Tel-Aviv University, Tel-Aviv 6997801, Israel; ⊥ ADAMA Center for Novel Delivery Systems in Crop Protection, Tel-Aviv University, Tel-Aviv 6997801, Israel

**Keywords:** steroids, hydrogels, PEG, controlled release, biodegradable, microneedles, in situ forming hydrogel, enzyme-responsive polymers, programmable mesophase transitions

## Abstract

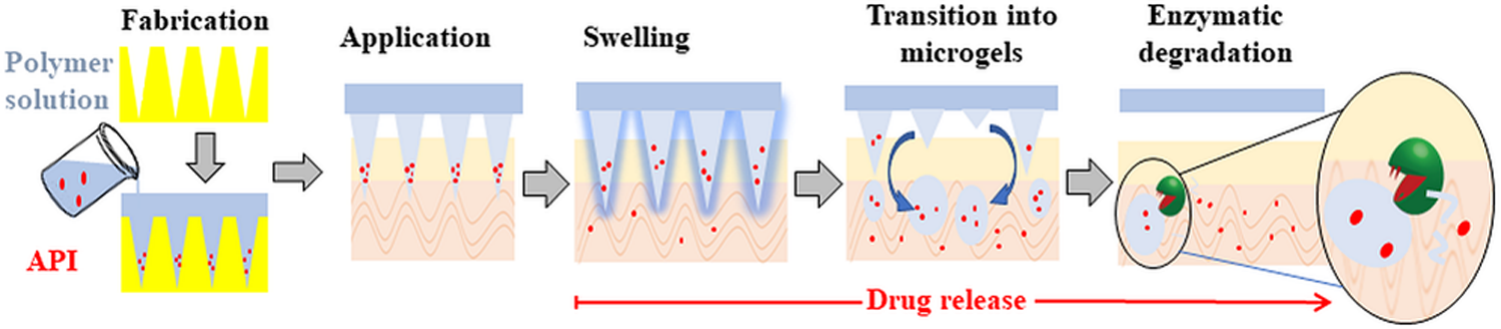

Microneedle-based drug delivery offers an attractive and minimally invasive administration route to deliver therapeutic agents through the skin by bypassing the stratum corneum, the main skin barrier. Recently, hydrogel-based microneedles have gained prominence for their exceptional ability to precisely control the release of their drug cargo. In this study, we investigated the feasibility of fabricating microneedles from triblock amphiphiles with linear poly(ethylene glycol) (PEG) as the hydrophilic middle block and two dendritic side-blocks with enzyme-cleavable hydrophobic end-groups. Due to the poor formation and brittleness of microneedles made from the neat amphiphile, we added a sodium alginate base layer and tested different polymeric excipients to enhance the mechanical strength of the microneedles. Following optimization, microneedles based on triblock amphiphiles were successfully fabricated and exhibited favorable insertion efficiency and low height reduction percentage when tested in Parafilm as a skin-simulant model. When tested against static forces ranging from 50 to 1000 g (4.9–98 mN/needle), the microneedles showed adequate mechanical strength with no fractures or broken segments. In buffer solution, the solid microneedles swelled into a hydrogel within about 30 s, followed by their rapid disintegration into small hydrogel particles. These hydrogel particles could undergo slow enzymatic degradation to soluble polymers. In vitro release study of dexamethasone (DEX), as a steroid model drug, showed first-order drug release, with 90% released within 6 days. Eventually, DEX-loaded MNs were subjected to an insertion test using chicken skin and showed full penetration. This study demonstrates the feasibility of programming hydrogel-forming microneedles to undergo several mesophase transitions and their potential application as a delivery system for self-administration, increased patient compliance, improved efficacy, and sustained drug release.

## 1 Introduction

There is a critical demand for advanced drug delivery systems to offer an efficient drug localization at targeted site and to circumvent the complications associated with drug delivery routes including the rapid metabolism of drug, nonspecific biodistribution, lack of patient adherence, and drug stability.^1,2^ Microneedles (MNs), which consist of an array of needles with heights ranging from 50 to 1500 μm, show promising prospects in transdermal drug delivery. MN-based delivery systems are capable of penetrating the skin barrier without stimulating nerve fibers, allowing a minimally invasive alternative to parenteral drug administration.^3−5^ MNs provide an efficient approach for drug penetration and an improved bioavailability, as they create micrometer-sized pores, allowing the transfer of drugs and macromolecules through the distinct layers of the skin. Hence, the use of MNs has the potential for broadening the spectrum of the drugs that can be delivered by conventional transdermal dosage forms, which are limited to drugs with molecular masses of less than 500 Da, a high partition coefficient, and high potency. Additionally, the utilization of MNs for drug delivery offers a strategic advantage by circumventing challenges associated with oral drug delivery within the gastrointestinal (GI) tract such as first-pass metabolism, enzymatic degradation, and GI irritation.^6−8^

Microneedles are classified into five different categories according to their design and drug delivery mechanism, namely, solid, hollow, coated, dissolving, and hydrogel-forming microneedles.^9^ Among these different types, hydrogel-forming (HF) MNs are an emerging type in which the hydrogel is formed in situ by swelling as a result of the uptake of skin interstitial fluid, leading to an increase in the dimensions of the resultant microchannels while maintaining physical and chemical structure integrity, and eventually allows for improved drug delivery into the skin.^10^ HF MNs are mainly fabricated from cross-linked polymers where altering the degree of cross-linking could be used to control the drug release, to provide long-term and continuous drug administration for improved therapeutic outcomes.^11−14^

Amphiphilic block copolymers have been utilized as building blocks in the development of nanocarriers due to their ability to self-assemble in aqueous solution, forming polymeric assemblies that can encapsulate an active ingredient inside the hydrophobic core and shield it from the biological environment.^15^ Moreover, due to the enhanced permeability and retention (EPR) effect, these assemblies are able to accumulate at the target site, such as inflamed or cancerous tissue, depending on their structural complexity and size.^2,16^ Furthermore, stimuli-responsive moieties can be incorporated to facilitate their disassembly and achieve targeted delivery.

Recently, stimuli-responsive polymeric MNs have attracted significant attention in the field of drug delivery. These MNs can release their payloads in response to endogenous or exogenous stimuli, such as pH, reactive oxygen species, light, temperature, enzymes, and mechanical forces. Such MNs can be used to deliver drugs in a more controllable manner, improve the therapeutic efficacy, reduce potential side effects, and provide a precise dosing.^17−19^

In the past decade, modular pathways were developed for the synthesis of polymeric amphiphiles based on dendrons with enzymatically cleavable end-groups. Using the high molecular precision, which emerges from the hydrophobic dendritic blocks, enzymatically degradable polymeric amphiphiles of various compositions, architectures, and hydrophilic-to-hydrophobic ratios were synthesized, and their self-assembly and enzymatic degradation were carefully characterized.^20−22^ These studies have resulted in a profound understanding of the structural parameters that govern the interaction of polymeric assemblies with enzymes. Based on these studies, the Amir group has recently expanded their studies toward the design and synthesis of triblock-based amphiphiles that were used to fabricate hydrogel-based microfibers by electrospinning,^23^ and even more recently, enzymatically degradable hydrogels.^24^

The unique architecture and high molecular precision of the triblock-based amphiphiles, together with their ability to form gel upon hydration and their enzymatic responsiveness, drove us to apply these features for the development of localized drug delivery platforms based on microneedles. These triblock-based MNs could be envisioned to undergo multistep mesophase transitions upon skin insertion to facilitate not only sustained drug release but also complete biodegradation and enhanced biocompatibility.

**Figure 1:**
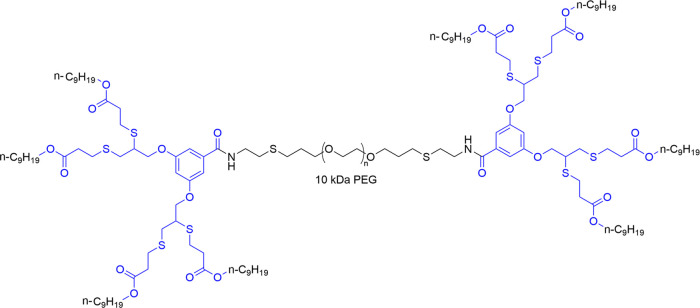
Synthesis of PEG-based triblock amphiphiles (Tri-C9) composed of hydrophobic dendrons containing esterase cleavable aliphatic end-groups.

Therefore, the purpose of this study is to demonstrate the ability to achieve controlled drug release by developing formulations for the fabrication of programmable microneedles. These MNs would transform into enzymatically degradable drug eluting hydrogels upon their penetration into the skin and absorption of interstitial fluid, followed by their in situ transition into gel microparticles, which can enhance drug release and be fully degraded by target enzymes into soluble hydrophilic polymers. Herein, as a proof of concept, the triblock polymeric amphiphile is composed of a central hydrophilic poly(ethylene glycol) (PEG, 10 kDa) block conjugated with two hydrophobic dendritic branching units functionalized with ester-based nonyl end-groups (Tri-C9), which can potentially be cleaved by skin esterases^25^ (Figure 1), and dexamethasone (DEX) was used as a model drug.

## 2 Materials and Methods

### 2.1 Materials

Dexamethasone (Alfa Aesar), sodium alginate (SA, Fisher Chemical), and trypan blue powder (Thermo Scientific) were purchased from Holland-Moran Inc., Israel. Silicone MPatch microneedle templates were purchased from Micropoint Technologies Pte Ltd. (Pioneer Junction, Singapore) and were pyramidal in shape with dimensions of 10 × 10 needle array, 200 μm base, 500 μm height, and 500 μm pitch. Organic solvents and PEG with average MWs of 3.4, 10, 35, and 100 kDa were purchased from Sigma Aldrich. Poly(vinyl pyrrolidone) (PVP) K-30 (MW 40 kDa) was purchased from BASF. Microcrystalline cellulose was supplied by FMC BioPolymer (Avicel, Philadelphia, PA), and PLGA-Purasorb PDLG 5010 (50:50) was donated by Corbion Purac (Gorinchem). Phosphate-buffered saline (PBS, pH 7.4) was purchased from Hyclone Laboratories. GeBaFlex tubes with a molecular weight cutoff (MWCO) of 8 kDa, porcine liver esterase (PLE), and bovine serum albumin (BSA, Probumin) were purchased from Merck.

### 2.2 Optimization Process of Tri-C9MNs Fabrication

MN arrays were prepared based on our previous work, using a modified vacuum-deposition casting method.^26^ Silicone MN molds were used to fabricate MN arrays of 10 × 10 pyramid-shaped microneedles, with 500 μm needle height, 200 μm base diameter, and interspacing of 500 μm. As a starting point, the feasibility of fabricating MNs made of an amphiphilic triblock polymer (Tri-C9) was investigated, and then, an optimization process was carried out to improve the characteristics of MNs (Table S1). In brief, the polymer was first dissolved in ethanol or chloroform to examine the effect of the organic solvent on film formation. Next, an aqueous solution of sodium alginate was added on top of the tip layer to form a base layer and enhance the polymer’s film-forming ability. As this step proved successful, the mechanical properties of the obtained MNs were assessed. To achieve the desired mechanical properties, different excipients were introduced into the formulation. The optimal formulation candidate was thereafter evaluated with different concentrations and molecular weights of the excipient.

### 2.3 Fabrication of PEG-Containing MN Arrays

**Scheme 1:**
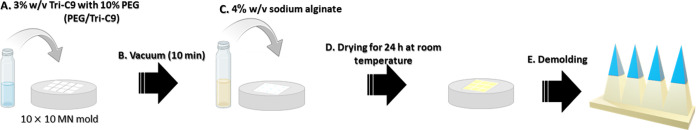
Schematic Illustration Showing the Process of Fabrication of Tri-C9MNs with an Alginate Base (A) Casting of the polymeric solution into polydimethylsiloxane (PDMS) mold to form the tips, (B) then the template was subjected to a vacuum for 10 min, (C) casting the baseplate-forming solution followed by (D) drying for 24 h, and (E) demolding of the MN array.

Based on preliminary findings, PEG was chosen for further investigations. As depicted in Scheme 1, polymeric solution of 3% w/v of amphiphilic triblock copolymer and 10% w/w (with regard to the triblock amphiphile) PEG with different molecular weights in chloroform was cast into the mold and degassed in a vacuum for 10 min to form the MN tips. Then, an aqueous solution of 4% w/v sodium alginate was added to the mold and allowed to dry overnight in a desiccator. After drying, the microneedle patches were detached from the mold and kept for further investigation. MN patches were characterized in terms of mechanical strength, insertion capability, and surface morphology.

### 2.4 Insertion Capabilities of MNs and Mechanical Property Testing

The insertion test was performed using eight stacked layers of Parafilm (∼140 μm each) as a skin simulant to investigate the penetration ability of the MN arrays as well as the insertion depth.^26−28^ This skin-simulant model was developed and validated by Larrañeta et al., showing that the insertion profiles obtained are consistent with the insertion depths obtained with optical coherence tomography (OCT), and the force that was used in this test gave insertion profiles equivalent to those obtained using neonatal pig skin.^27^ Briefly, the obtained MNs were inserted manually into the Parafilm layers, held for 30 s, and then removed. The number of holes created in each layer and the height of the MNs after insertion were evaluated using a stereomicroscope (Olympus-SZ61, Tokyo, Japan). To further evaluate the mechanical strength of the MNs, they were positioned in an upright manner and then underwent compression with forces ranging from 50 to 1000 g for 5 min; subsequently, morphological and dimensional changes of the needles were evaluated.^29^

### 2.5 Fabrication and Characterization of DEX-Loaded MNs

Based on the aforementioned characterization methods, the lead formulations were chosen for incorporating DEX as a model drug. DEX-loaded MN were prepared in a similar way as mentioned in Section 2.3, by adding 1 mg of DEX to the tip solution (final concentration 1% w/v). MN arrays were characterized by evaluating their penetration efficiency, surface morphology, and drug content. To determine the encapsulation efficiency (EE) and drug loading, DEX-loaded MN arrays were immersed in distilled water with continuous stirring to disintegrate the MN patch completely. Subsequently, water was evaporated by using a rotary evaporator. After that, 5 mL of acetonitrile was added to the resulting residue, and after serial dilutions, DEX was quantified using high-performance liquid chromatography (HPLC). All measurements were recorded on a Waters Alliance e2695 separations module equipped with a Waters 2998 photodiode array detector. Chromatographic separation was obtained using XBridge Protein BEH, C4, 3.5 μm, 4.6 mm × 150 mm column and a mobile phase consisted of water and acetonitrile (5:95) with a flow rate of 1 mL/min, the samples analysis time was 15 min, and the column temperature was maintained at 37° with an injection volume of 30 μL. DEX was detected at 241.5 nm (Figure S1).^30,31^

Morphological characterization of the MNs upon the incorporation of the drug was carried out using a Quanta 200 FEG environmental scanning electron microscope (SEM) in high vacuum (WD ∼ 10 cm, 3–20 kV). Before imaging, a thin layer of palladium (Pd) was deposited onto the needle arrays to ensure better visualization. In addition, to elucidate the polymer disposition within the lead MN formulation, an elemental analysis was performed using energy dispersive spectroscopy (EDS) on a separated single tip and baseplate of the MN array.

### 2.6 Swelling Studies of Hydrogel-Forming MN in PBS Solution

In order to better visualize the swelling of MNs, curcumin (1 mg) was incorporated into the Tri-C9MN patch with 35 kDa PEG using the same preparation procedure as above. The tips of the MNs were placed on a supporting Parafilm platform to ensure exposure of the MNs tips, where the triblock amphiphilic polymer is mainly located, to the aqueous media. Afterward, PBS solution (pH 7.4) was added, and the transition stages of the tips were visualized during the test using a HAYEAR 4K UHD microscope camera.

### 2.7 In Vitro Drug Release Studies

The in vitro release of DEX from the hydrogel-forming MN arrays was determined using PBS (pH 7.4) solution containing 1 mg/mL bovine serum albumin (BSA), which exhibits an affinity to hydrophobic entities.^32−34^ Porcine liver esterase (PLE) was used as a model for esterase activity. Briefly, Tri-C9MNs with 10% w/w 35 kDa were placed in GeBaFlex tubes (8 MWCO kDa) containing 0, 15, or 45 μM of PLE enzyme, and then the tube was immersed in 40 mL of the release medium (PBS containing BSA). At predefined points, 0.5 mL of the release medium was sampled and replaced with 0.5 mL of fresh prewarmed release medium to maintain the sink conditions. The samples were analyzed by using HPLC with UV detection at 241.5 nm. At the end of the experiment, acetonitrile was added to the samples inside the dialysis tube to fully dissolve them, and the obtained solutions were injected to HPLC with UV detection at 293 nm to analyze the degree of degradation of the Tri-C9.

### 2.8 Ex Vivo Skin Insertion Test of DEX-Loaded MNs

DEX-loaded Tri-C9MNs (35 kDa PEG) were evaluated for their ability to penetrate chicken skin (obtained from a local slaughterhouse). A tension/compression tester consisting of a motorized vertical test stand equipped with a digital force gauge (PCE-VTS 50-DFG N 500) was employed (PCE Instruments UK Ltd., Manchester, U.K.). In this test, the MN samples were glued to the moving probe of the machine and were pressed against fixed chicken skin at a rate of 1 mm/min, until the desired normal force of 20 N was achieved (on average 200 mN/needle), followed by 30 s waiting dwell time under the loaded state, followed by withdrawing the upper holder that holds the MN samples in the opposite normal direction.^11,14,35,36^ Continuous force and displacement measurements were recorded to identify the point of needle insertion. The curves of force versus displacement were generated for each test, and the average insertion force was determined from 5 independent measurements. After MN array removal, the skin area, where the MNs were inserted, was inspected using a HAYEAR 4K UHD microscope camera. To better visualize the insertion area, the skin was stained with 0.4% w/v trypan blue aqueous solution (left for 5 min, after which the excess of trypan blue was rinsed with ethanol and wiped away).^37^

## 3 Results and Discussion

### 3.1 Optimization Process of Tri-C9MNs

The first step was to demonstrate the fabrication of MN from the dendritic triblock amphiphile. As a starting point, ethanol and chloroform were used to cast the Tri-C9 polymer; however, aggregates were formed instead of a homogeneous MN array (Figure S2); this is attributed to the swift solvent evaporation rate and the fragility of the polymer film, wherein the polymer chains have limited opportunities to entangle, hindering the formation of a stable film layer on the MN mold.^38^ Consequently, poly(vinyl pyrrolidone) (PVP) was incorporated into the polymeric solution in ethanol, due to its film-strengthening ability.^39,40^ Upon drying, the MN array was detachable, yet the resultant MNs showed poorly defined shape, nonhomogeneous spreading of the polymer, and incomplete filling of the MN mold cavities (Figure S3). Considering these findings and based on our previous study,^26,28^ the baseplate was prepared using an aqueous solution containing SA to allow easy demolding and a more definite structure. The rationale behind this step is the fact that although the Tri-C9 polymer does not dissolve in water, it can swell into a hydrogel mesophase; therefore, the addition of SA aqueous solution should further result in further penetration of the polymer into the needle cavities of the mold. Furthermore, with the evaporation of water and increase in the relative concentration of the polymers, the SA chains can interpenetrate the Tri-C9 network to form a continuous matrix with increased stability, while the organic-based polymeric solution exhibited negligible viscosity, which led to poor film-forming ability.^41^

Next, due to the limited solubility of the Tri-C9 polymer in ethanol, its optimal concentration in chloroform was investigated. Tri-C9 polymeric solutions with concentrations of 3, 5, and 10% (w/v) in chloroform were cast into the MN molds to form the needles, and SA aqueous solution was added to form the baseplate. Increasing the polymer concentration led to a decrease in the capability of MN formation (Figure S4). High concentrations (5 and 10%) of the Tri-C9 polymer resulted in aggregates deposited in the mold cavities that prevent the SA solution from being homogeneously spread over the template. Therefore, a concentration of 3% w/v was chosen as the optimal Tri-C9 concentration for further investigation.

To get an insight into the mechanical strength of the MNs, they were inserted manually into the skin-simulant Parafilm model.^27^ Significant bending of the needles was observed, indicating poor mechanical properties. Therefore, several excipients were examined with the aim of increasing the MNs’ mechanical strength. Initially, poly(lactic-*co*-glycolic acid) (PLGA), a common copolymer with good mechanical properties,^42−44^ was incorporated into the MN formulation, by either combining the Tri-C9 polymer with PLGA at the same ratio and casting into the MN mold or by forming trilayer MN by casting the PLGA into the tips followed by casting the Tri-C9 polymer solution and then the baseplate as stated earlier. While PLGA improved the insertion capability of the MNs in Parafilm, a phase separation between the two polymers was noticed, suggesting incompatibility between them (Figure S5). Afterward, microcrystalline cellulose (MCC), a widely used compression excipient serving as a binder in pharmaceutical dosage forms,^45^ was examined but the obtained MNs still showed inadequate mechanical properties (Figure S6). MCC did not integrate effectively into the MN array, presumably due to its lower affinity to chloroform, in comparison to the Tri-C9 polymer, leading to decreased compatibility between them. Additionally, MCC exhibits strong molecular interaction with water, leading to the diffusion of MCC into the film surface upon addition of the aqueous sodium alginate layer.^46^

Lastly, PEG with a molecular weight of 3.4 kDa was tested due to its potential to increase the entanglement of the polymer chains.^23^ PEG is often used as a binder in various drug formulations such as tablet manufacturing, in which it acts as an adhesive to bind the granules and other additives together.^47,48^ Two concentrations of PEG were used: 5 and 10% w/w PEG/Tri-C9. The incorporation of PEG significantly improved the MN arrays in terms of structural integrity, uniformity, and mechanical strength, as confirmed by the insertion test using Parafilm layers, providing vital indication for meeting the basic mechanical requirements of MNs (Figure S7).

### 3.2 Fabrication and Characterization of Tri-C9MNs with PEG

**Figure 2:**
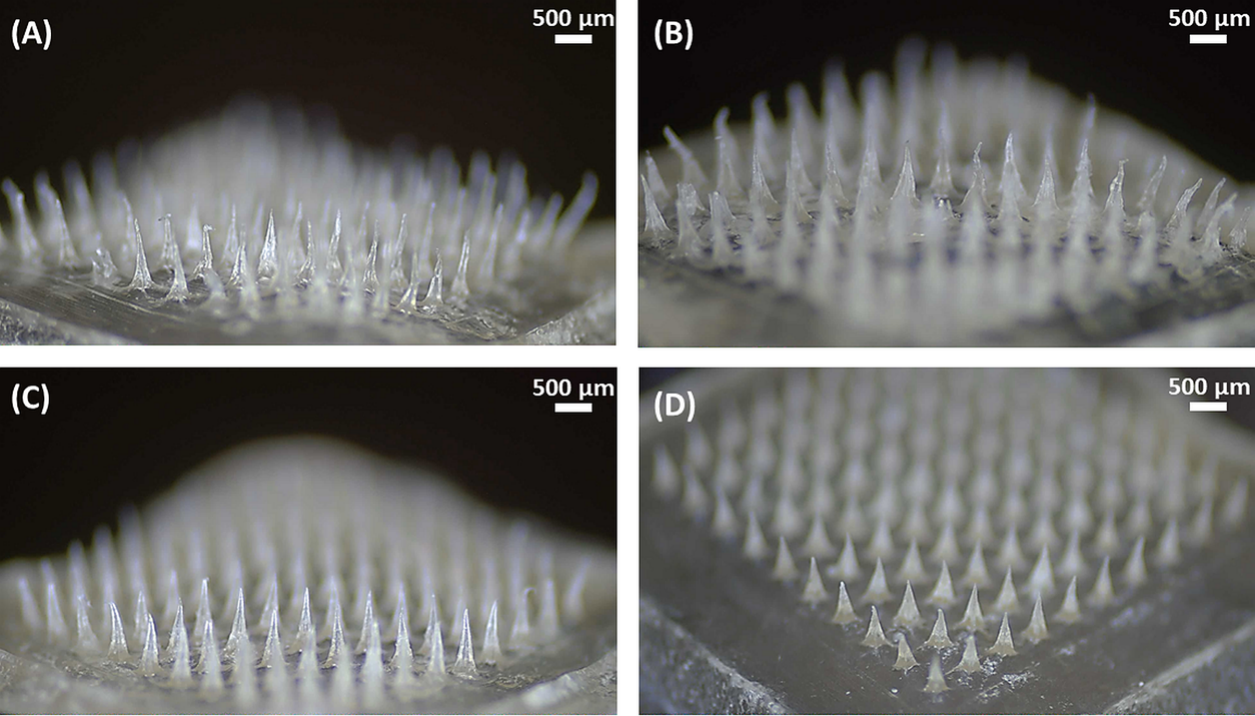
Representative images of the resultant MN patches with 10% w/w PEG/Tri-C9: (A) 3.4 kDa PEG, (B) 10 kDa PEG, (C) 35 kDa PEG, and (D) 100 kDa PEG.

Based on the obtained findings, PEG was selected as the leading excipient and was integrated into the MNs at a concentration of 10% w/w PEG/Tri-C9. Next, MN preparations with varying molecular weights of PEG (3.4 10, 35, and 100 kDa) were investigated as candidate formulations. The fabricated MN patches consisted of an array of 10 × 10 pyramidal needles with a base width and height of 200 and 500 μm, respectively. Tri-C9 polymer and PEG were mainly concentrated at the tips of the needles, while the baseplate consisted of sodium alginate. As depicted in Figure 2, Tri-C9MNs made with 3.4 and 10 kDa PEG showed bent-shaped needles and incomplete tips, while MNs formed with 35 and 100 kDa PEG demonstrated well-defined sharp tips and uniform distribution on the substrate, confirming that the molecular weight of PEG affects both the physical appearance and mechanical properties of Tri-C9MNs. This can be attributed to the ability of the higher molecular weights of PEG to contribute to increased stiffness, while the addition of intermediate molecular weight has a plasticizing effect on the MN patches as they adversely affect the structural properties and flexibility.^49,50^

### 3.3 Insertion Capabilities of MNs in the Skin-Simulant Model and Mechanical Property Testing

The mechanical strength of MNs as drug carriers is an essential consideration due to the crucial demand for MNs to have an adequate strength capable of piercing the foremost transdermal barrier, the stratum corneum.^51^ Thus, the mechanical performance and insertion testing of MNs with PEG with different molecular weights were evaluated as previously described by Larrañeta et al. for which Parafilm was used as a skin simulant.^27^ For this aim, eight layers of Parafilm were assembled to create a film with an approximate thickness of 1 mm, and the MNs were inserted by manual pressure to imitate the practical use in clinical settings.

**Figure 3:**
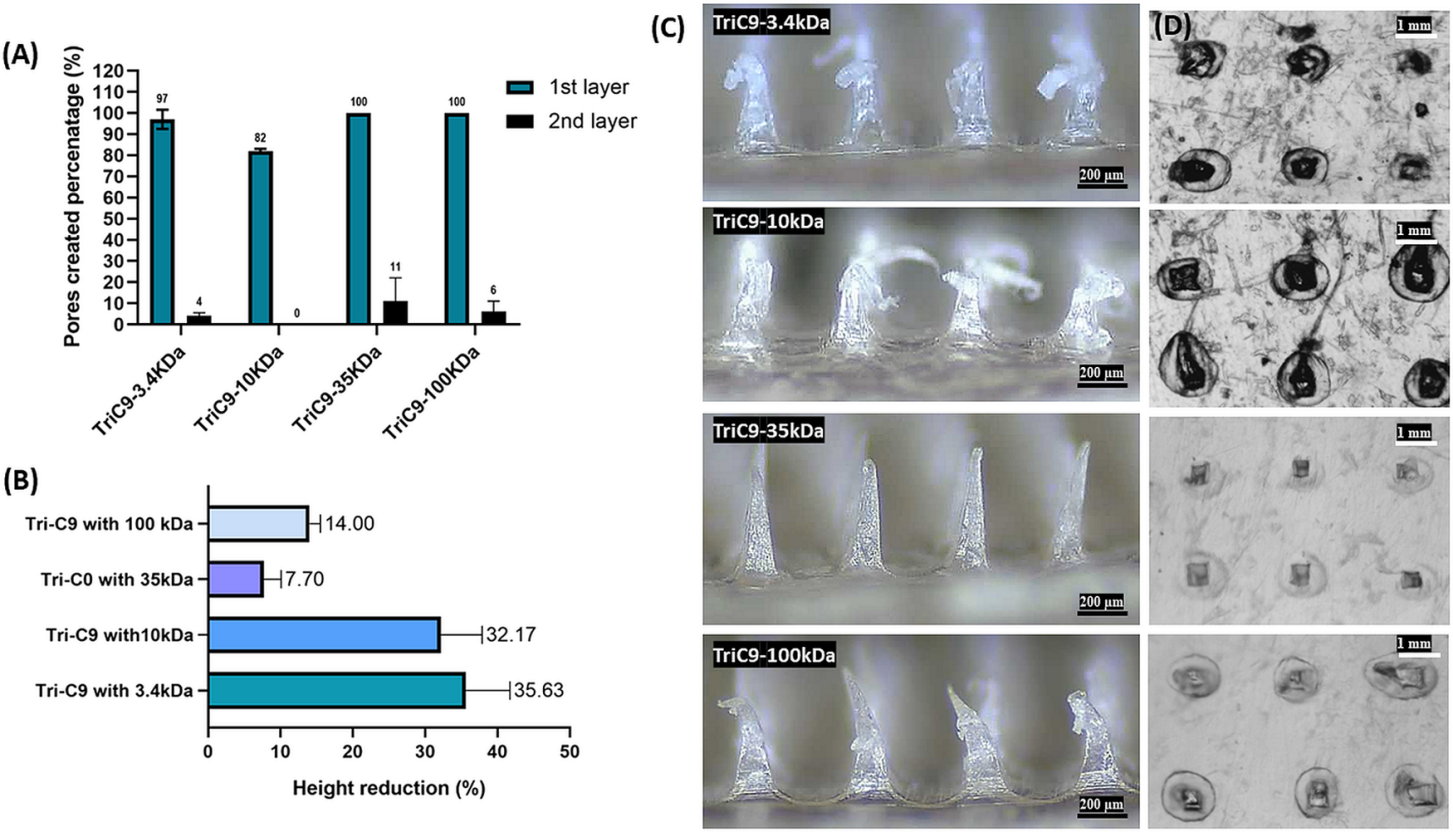
Insertion test in the Parafilm skin-simulant model. (A) Percentage of holes created in the first two Parafilm layers by the MNs. (B) Height of MNs post insertion to Parafilm layers. Results are expressed as means ± s.d., *n* = 10. (C) MNs bending after insertion into the Parafilm layer and (D) the holes observed on the first layer by each MN patch.

Tri-C9MNs with 35 and 100 kDa PEG exhibited complete perforation of the first Parafilm layer and a number of holes created in the second layer, whereas Tri-C9MNs with 3.4 and 10 kDa PEG displayed lower penetration ability accompanied by higher height reduction percentage, indicating poor mechanical performance of the latter (Figure 3). In this regard, Tri-C9MNs with 35 kDa PEG created well-defined square-shaped pores in the Parafilm and were slightly compressed rather than bent, demonstrating their mechanical adequacy and uniformity. On the other hand, the remaining MN formulations were significantly bent (Figure 3C).

**Figure 4:**
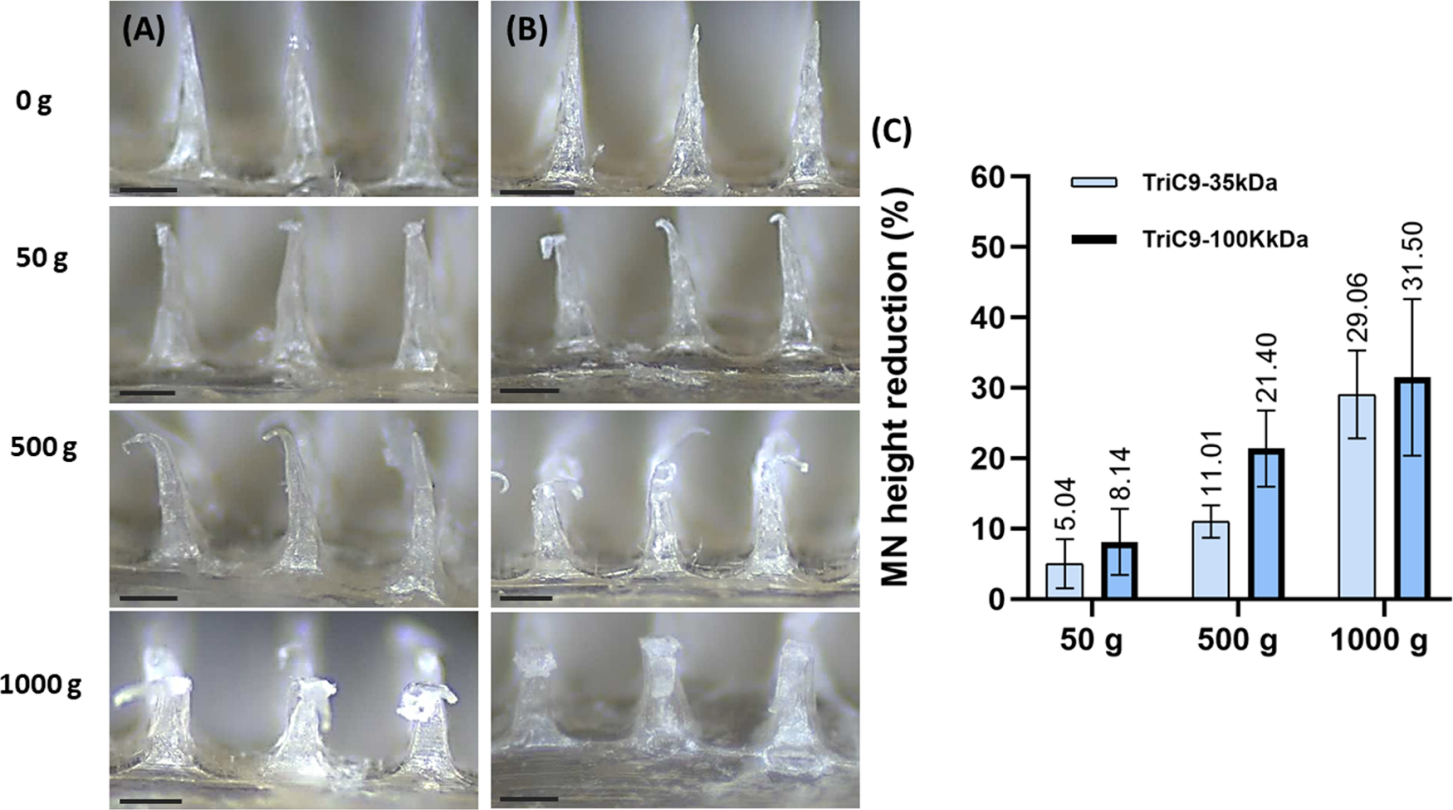
Evaluation of the mechanical strength against static force. Morphological changes of (A) 35 kDa/Tri-C9MNs and (B) 100 kDa/Tri-C9MNs after placement of 0, 50, 500, and 1000 g. (C) Percentage of MN height reduction after compression; results are expressed as means ± s.d., *n* = 10.

To further shed light on the mechanical strength of the lead MN formulations, with 35 and 100 kDa PEG, the resistance of MNs to increasing static forces was measured by placing different weights on the top of the MN arrays for 5 min. As displayed in Figure 4, the MNs underwent deformation that can be clearly observed when compared to the original state, in which the sharp tips of MNs presented more and more bending after putting with 50 g (∼4.9 mN/needle) to 1000 g (∼98 mN/needle) weights on MNs. However, the MNs remained intact and did not break, indicating their good mechanical strength and potential competency for transdermal drug delivery.^26,52,53^

Taken together, incorporating PEG with high molecular weights into the MN formulations led to an improvement in the mechanical properties due to the increased polymeric chain density at the MN tips.^26,54^ Furthermore, PEG was previously found to increase the entanglement of the polymeric chains,^23^ and this was further reinforced by the analysis of the thermal behavior of the polymer blend of the triblock polymer and PEG 35 kDa using differential scanning calorimetry (DSC). Figure S8 illustrates the increase in the melting point of the polymer; a higher melting point referred to more intertwined polymer chains and hence for strong intermolecular interactions.^50^ Based on the skin-simulant model results and observed mechanical properties, Tri-C9 with 35 and 100 kDa PEG were selected as lead formulations for further investigation.

### 3.4 Preparation and Characterization of DEX-Loaded MNs

The anti-inflammatory drug, DEX, was used as a lipophilic model drug and was successfully incorporated into the needle tips of Tri-C9MNs with 35 and 100 kDa PEG. Both formulations displayed comparable encapsulation efficiencies with insignificant difference (*t* test, *p* > 0.05) and drug-loading content of ca. 8.5 wt % (Table 1). The pyramidal morphology of the MN arrays was confirmed by using scanning electron microscopy (SEM).

**Table 1:** Encapsulation Efficiency and Drug-Loaded Content of Tri-C9MNs with Different Molecular Weights of PEG (*n* = 3, Means ± s.d.)

	Tri-C9MN with 35 kDa PEG	Tri-C9MN with 100 kDa PEG
encapsulation efficiency (%)	87 ± 6	88 ± 3
loading content (%)	8.5 ± 0.5	8.6 ± 0.2

**Figure 5:**
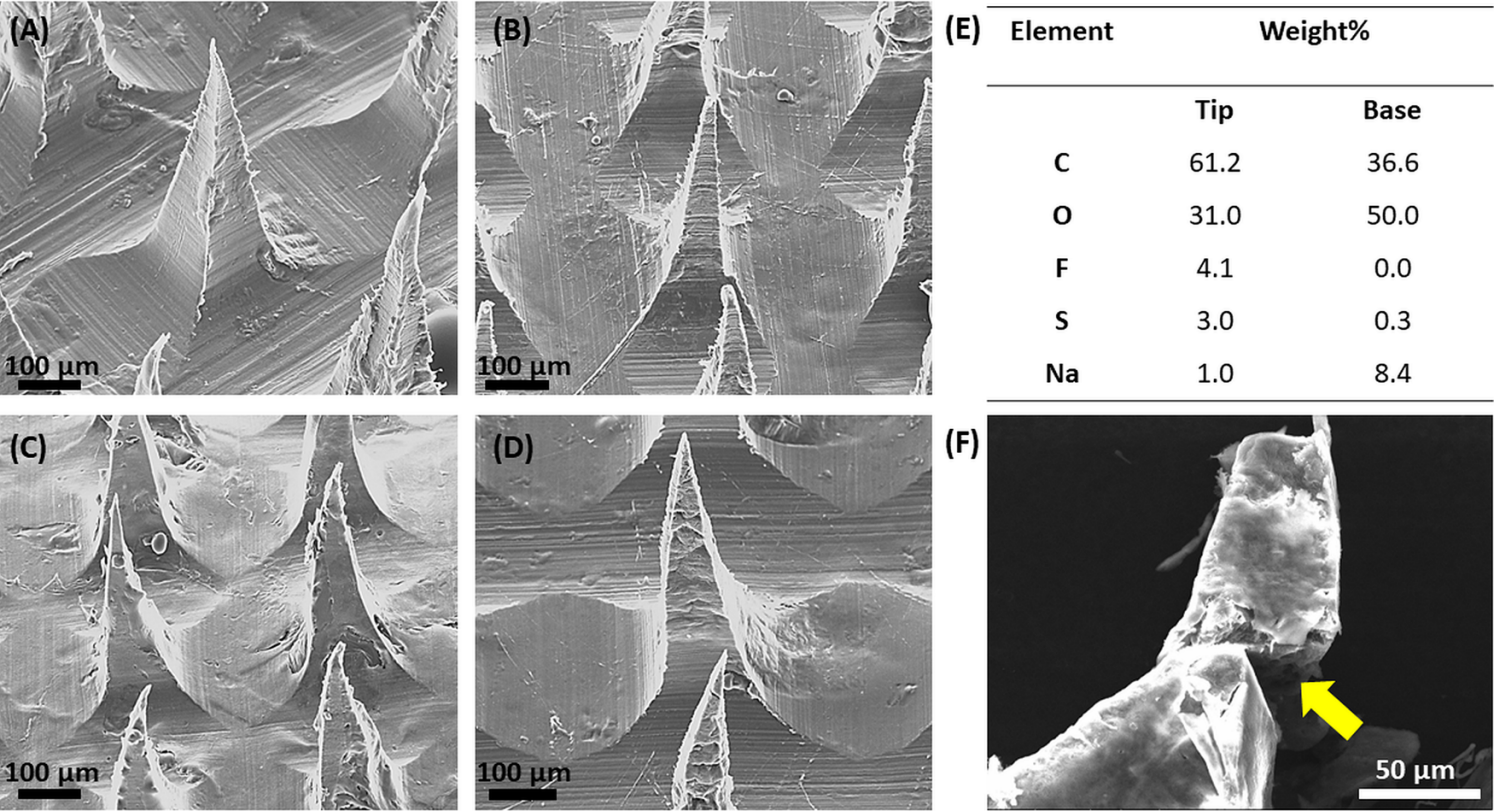
SEM images of (A) blank Tri-C9MNs with 35 kDa PEG, (B) DEX-loaded, (C) blank Tri-C9MNs with 100 kDa PEG, and (D) DEX-loaded MNs. Scale bar: 100 μm. (E) Elemental composition: the weight percentage of the elements detected in the tested sample and (F) SEM image of a fractured tip and yellow arrow pointing to the area analyzed by EDS.

Figure 5A–D shows drug-loaded Tri-C9MNs containing 35 and 100 kDa PEG, compared to their blank counterparts. All formulations had intact structures with a quadrangular pyramidal shapes indicating that the MNs can be fabricated from the amphiphilic triblock polymer and successfully load the drug. To better describe the composition of the MN tips and baseplate, an elemental analysis was performed using energy dispersive spectrometry (EDS). While carbon and oxygen are indicatives of both Tri-C9 polymer and sodium alginate, higher carbon-to-oxygen ratio is expected for the Tri-C9 polymer. In addition, while sodium should be characteristic of the sodium alginate baseplate, sulfur is derived only from the Tri-C9 polymer, and the drug is the only source of fluorine. Therefore, we studied the distribution of carbon, oxygen, fluorine, sodium, and sulfur to gain further insight into the arrangement of the constituent materials. Figure 5E summarizes the quantitative contents of the constituent materials. Analysis of the tip of a single needle (Figure 5F) showed a significantly higher carbon-to-oxygen ratio in comparison with the baseplate (Figure S9), which was found to contain a higher oxygen-to-carbon ratio. In addition, the tip part was also found to contain higher amount of sulfur than sodium. Conversely, when analyzing the baseplate, the amount of sodium surpassed that of sulfur. This suggests that the polymer predominantly positioned in the upper part of the MN, while sodium alginate is primarily located at the baseplate of the microneedle patch. Additionally, a fluorine atom signal was observed only in the tip analysis and was absent in the baseplate, confirming the successful encapsulation of the drug within the polymer in the tips of the MNs.

The drug-loaded MN patches were evaluated in terms of their insertion ability by conducting a Parafilm insertion test. Both DEX-loaded Tri-C9MNs created more than 95% in the first Parafilm layer, equivalent to ca. 140 μm of insertion depth, demonstrating their ability to deliver the drug into the epidermis layer by penetrating the stratum corneum. The MN height reduction percentage was calculated after the insertion test for each formulation in which DEX-loaded MNs with 35 kDa PEG exhibited a height reduction of 23 ± 8%, whereas DEX-loaded MNs with 100 kDa PEG showed 29 ± 6% height reduction percentage. Although the MNs displayed greater deformation than in the absence of the drug (Figure 3), no evidence of breakage or fractures was observed (Figure S10). It can be concluded that both the molecular weight of PEG and the drug loading could significantly influence the mechanical properties of the Tri-C9MNs. Generally, the mechanical strength of MN arrays is widely affected by several factors including polymer type and concentration, encapsulated drug type and concentration, and fabrication methods.^5,55^ Our results are in agreement with previous studies, which reported the weakening of the mechanical strength of MNs by loading them with drug molecules. Park et al. demonstrated that the incorporation of calcein into MNs made of PLGA led to a remarkable reduction in the mechanical strength of the MNs. The authors indicated that the polymer matrix and the poor adhesion between the drug and polymer could have a primary role in creating mechanical failure sites for the MNs.^56^ Du et al. investigated the mechanical properties of two types of hyaluronic acid MNs with different molecular weights, with or without loaded model drugs, lidocaine hydrochloride and bupivacaine hydrochloride. The results exhibited that both the molecular weight of the polymer and the loading of drug could significantly influence the mechanical properties of MNs, where both model drugs significantly decreased them.^57^

Regarding the difference between the mechanical performance of MN arrays with 35 kDa PEG and 100 kDa PEG, while the molecular weight of the polymer is one of the significant factors that contribute to its mechanical strength, it is not the only one. It is well-known that the strength of polymers increases with size until a critical molecular weight, beyond which there is no longer correlation between mechanical properties and molecular weight.^58−61^ Based on the aforementioned findings, we selected Tri-C9 with a 35 kDa PEG formulation for further investigations.

### 3.5 MN Arrays’ Behavior upon Exposure to an Aqueous Medium

**Figure 6:**
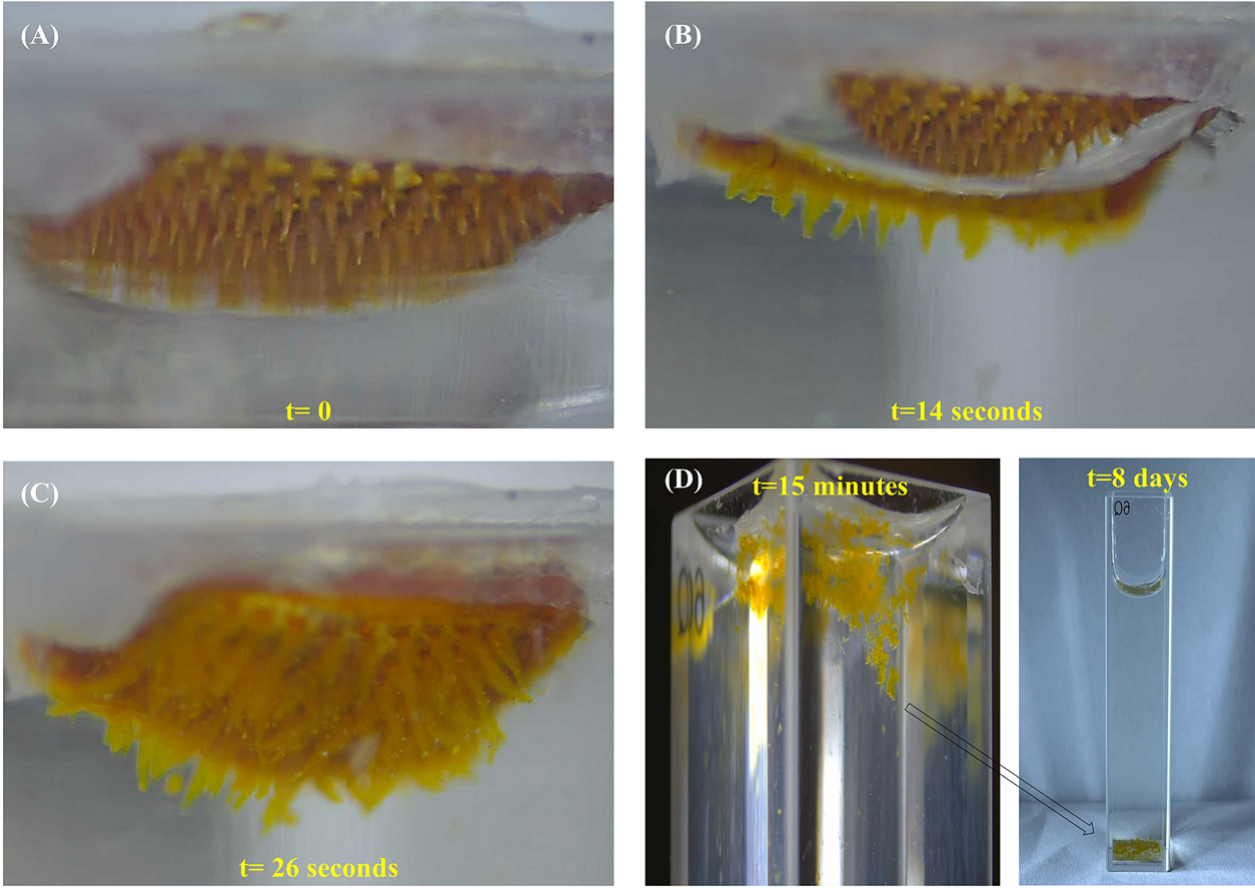
Digital microscopic view of MN tips upon exposure to PBS solution (pH = 7.4): (A) *t* = 0, (B) *t* = 14 s, (C) *t* = 26 s, and (D) images of the aqueous medium containing the obtained hydrogel after 15 min and 8 days.

To assess the potential ability of the MNs to imbibe the interstitial fluids of the body and undergo mesophase transitions, we examined their swelling capacity and dynamic transition stages in phosphate-buffered saline. Upon introduction into PBS, the wetting process of MN involved two main stages, which included dissolving and swelling phases that occurred simultaneously. The first stage includes rapid dissolving of the hydrophilic PEG excipient and the sodium alginate as a result of the interaction with water molecules, while in the second stage, the amphiphilic triblock absorbs water and swells into a hydrogel, due to the existence of the hydrophobic dendrons. As depicted in Figure 6 and Movie S1, the MN patch swells rapidly, and subsequently, a third stage of disintegration of the formed hydrogel can be observed within 30 s. In this stage, the swollen hydrogel needles started to disintegrate and formed microgel particles, which gradually precipitated to the bottom of the container. The hydrogel particles that were formed remained stable, even after 1 week.

### 3.6 In Vitro Drug Release from MN Tips

**Figure 7:**
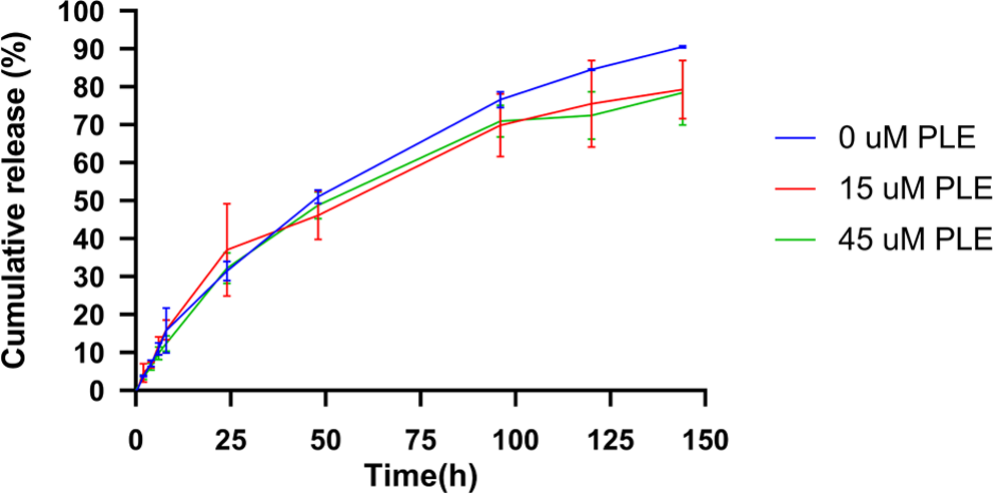
In vitro release of dexamethasone from Tri-C9MNs (pH 7.4, 37 °C). The values are means ± s.d. of three experiments.

Encouraged by the multistep transition from solid MNs to swollen hydrogel MNs and then to hydrogel particles, next, we set to examine the release of dexamethasone from Tri-C9 with 35 kDa PEG-based MNs. DEX-loaded MNs were placed into a dialysis tube (MWCO 8 kDa) with an aqueous buffer (PBS, pH 7.4) in the presence or absence of an esterase to evaluate the release profile of the hydrophobic cargo upon the several transition mesophases of the Tri-C9 amphiphiles. Both conditions exhibited controlled release with rather similar release rates in the first 48 h. As presented in Figure 7, in the absence of PLE, approximately 90% of the drug was released within 6 days, while in the presence of the enzyme, the formulation exhibited a slightly decelerated release rate, resulting in a release of around 80% within the same time frame.

The data acquired from the drug release study were analyzed using zero-order, first-order, and Korsmeyer–Peppas release kinetic models in order to shed light on the release mechanism. Table 2 presents the correlation coefficient (*R*^2^) values for these kinetic models, showing that the first-order release and Korsmeyer–Peppas model provided the best fit for the data.

**Table 2:** Correlation Coefficient (*R*^2^) Values of Various Release Kinetic Models for DEX-Loaded MNs under Different Conditions (PLE Concentrations)

	zero order	first order	Korsmeyer–Peppas model	
condition	*R*^2^	*R*^2^	*n*	*R*^2^
without PLE	0.958	0.998	0.812	0.986
15 μM PLE	0.930	0.988	0.771	0.973
45 μM PLE	0.932	0.985	0.846	0.995

The Korsmeyer–Peppas kinetic model describes the drug transport mechanism by fitting the first drug release data (below 60% release) and calculating the value of the release exponent (*n*). Using DEX-loaded MNs, the release exponents under different PLE concentrations ranged from 0.77 to 0.84, indicating non-Fickian or anomalous transport. This suggests that the mechanism of DEX release is mainly governed by swelling and diffusion, where the slow rearrangement of polymeric chains and the diffusion of the drug, simultaneously cause the time-dependent anomalous effects.^62,63^ Moreover, the atypical geometry of the MNs could potentially also account for this drug release behavior. This can be attributed to certain physicochemical processes that have not been taken into account within the mathematical model applied, as it was not specifically tailored to accommodate these unique geometrical configurations.^64,65^

It is important to note that although the Tri-C9 amphiphiles contain aliphatic end-groups linked by ester bonds to the dendritic branches, we did not observe any significant difference in the release kinetics with or without the enzyme. This can be explained by considering our recent report on the slow enzymatic degradation of hydrogels composed of triblock amphiphiles with similar architecture and even lower degree of hydrophobicity (based on C6 chains).^66^ As the current release experiment shows that DEX release was faster than the expected enzymatic degradation, one can expect similar release rates regardless of the presence of the enzyme and its concentration. Moreover, as dexamethasone is a moderately lipophilic drug (log *P* 1.83),^67^ it can be expected to readily diffuse to the release medium so that the potential contribution of the hydrolysis of the amphiphiles by the enzyme becomes even more limited.^68^ Nevertheless, as eventual degradation of the amphiphiles can be a critical requirement to allow their clearance after releasing their cargo, we analyzed the degree of the degradation of the amphiphiles at the end of the release experiment. To do so, the solutions inside the dialysis tubes were diluted with acetonitrile to allow complete dissolution of the polymer residues and then analyzed by HPLC. The chromatograms (Figure S11) clearly showed full degradation of the amphiphiles into hydrophilic polymers for the samples that were incubated with the enzyme, while the samples without the enzyme were found to show only partially degraded triblock amphiphiles (due to spontaneous hydrolysis of the esters). When taking into account both the release rates and the HPLC analysis of the degree of degradation of the amphiphiles, the sustained release of dexamethasone can mainly contribute to the successful entrapment within the amphiphilic polymer matrix, underlining the exceptional potential of the designed triblock amphiphile-based MNs for controlled and prolonged drug release. Such sustained release behavior could increase the patient’s adherence to the treatment by reducing the need for frequent dose administration as well as minimizing the side effects often associated with higher doses.^69^

### 3.7 Ex Vivo Skin Insertion Test

**Figure 8:**
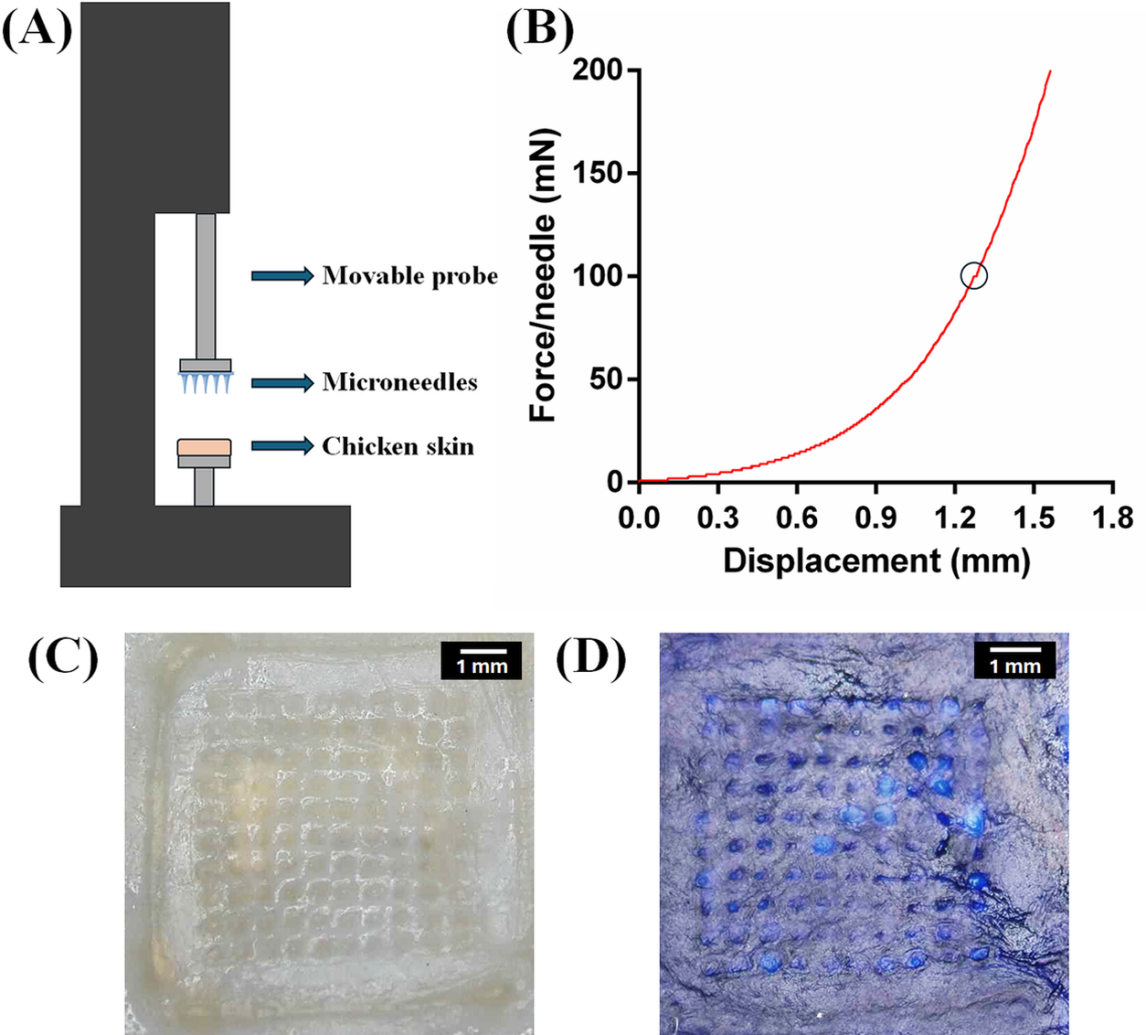
Skin insertion test of DEX-loaded Tri-C9MNs (35 kDa PEG). (A) Schematic representation of the compression tester setup for the determination of insertion force of MNs using ex vivo chicken skin. (B) Representative force–displacement curves of the MNs pressed against chicken skin. The point of insertion exhibits small a plateau marked by a circle. (C) Top view of chicken skin after MN insertion and (D) after staining by trypan blue.

To further elucidate the mechanical properties and insertion capability of DEX-loaded MNs, the MNs were subjected to penetration testing using ex vivo chicken skin (Figure 8A). Chicken skin has been employed as a suitable simulant for human skin by numerous researchers to characterize microneedle arrays.^28,70−72^ Skin penetration is characterized as a series of sequential small penetrations where the MNs gradually tear the skin, while the force increases until it reaches a plateau followed by more rapid increase, indicating successful skin piercing.^73−75^Figure 8B shows the force–displacement curve in which the force was normalized by the number of needles (force per a single needle) in each MN array. At the insertion point, an abrupt change in slope in the form of a small plateau is evident, and then while MNs being further inserted in the skin, the resisting force increases again due to the friction between the needles and the skin tissues, as well as the compression of the skin–MN system.^76^ The DEX-loaded MNs required a mean insertion force of 101 ± 1 mN/needle (*n* = 5). The insertion was confirmed by visualizing the skin after the compression test. The MNs demonstrated full penetration into the skin, as shown in Figure 8C. To improve the visibility of the puncture sites, the skin was stained with trypan blue solution, and blue pinholes were observed on it, indicating that the MNs had sufficient mechanical strength to successfully penetrate the skin (Figure 8D).

## 4 Conclusions

This study demonstrates the feasibility of using triblock amphiphiles to fabricate microneedles that can be programmed to undergo sequential mesophase transitions. We show that these microneedles can transform from a solid structure into hydrogel-based microneedles upon absorbing water. Subsequently, they undergo an in situ transition into hydrogel microparticles that can further undergo enzymatic degradation into soluble polymers.

We also showcase the potential of these microneedles for controlled drug release by loading them with dexamethasone as a model drug and monitoring the release kinetics. This programmable microneedle-based drug delivery system has the potential to be employed for controlled drug release in the upper skin layers. The programmable mesophase transitions can be applied to achieve local and more effective treatment by increasing the residence time of the drug at the target site, enabling better penetration and sustainable delivery for an extended period of time, while minimizing side effects.

## Supporting Information Available

Detailed design and characterization of different Tri-C9MNs prepared during the optimization step including compositions, tested parameters, representative images of the obtained MN arrays, results of insertion test in the Parafilm skin-simulant model; SEM/EDS of a single MN tip of DEX-loaded MNs with 35 kDa PEG; HPLC chromatograms of dexamethasone and the gel formed in the release experiment, as well as the conditions of HPLC analysis (PDF)
MN patch swells rapidly and subsequently a third stage of disintegration of the formed hydrogel can be observed within 30 s (MPG)

## Supplementary Material

mt3c01133_si_001.pdf

mt3c01133_si_002.mpg
