# Supplementary material for: Hydrogel Microneedles with Programmed Mesophase Transitions for Controlled Drug Delivery
Source: ACS Appl Bio Mater. 2024 Feb 9;7(3):1682–93. doi: 10.1021/acsabm.3c01133 (PMC10951948; doi:10.1021/acsabm.3c01133)
Supplement: Supplementary file 1 — mt3c01133_si_001.pdf [file mt3c01133_si_001.pdf]

Supporting Information

Hydrogel Microneedles with Programmed Mesophase Transitions for Controlled Drug Delivery

Hala **Dawud**[a], Nicole **Edelstein-Pardo**[b,c,d], Keerthana **Mulamukkil**[b,c,d], Roey J. **Amir**[b,c,d,e,]*, and Aiman **Abu Ammar**[a,]*

[a] Department of Pharmaceutical Engineering, Azrieli College of Engineering Jerusalem, Jerusalem 9103501, Israel

[b] School of Chemistry, Faculty of Exact Sciences, Tel-Aviv University, Tel-Aviv 6997801, Israel

[c] The Center for Physics and Chemistry of Living Systems, Tel-Aviv University, Tel Aviv 6997801, Israel

[d] The Center for Nanoscience and Nanotechnology, Tel-Aviv University, Tel Aviv 6997801, Israel

[e] ADAMA Center for Novel Delivery Systems in Crop Protection, Tel-Aviv University, Tel Aviv 6997801, Israel

* **Corresponding authors:**

amirroey@tauex.tau.ac.il, ORCID: https://orcid.org/0000-0002-8502-3302

aimanab@jce.ac.il, ORCID: https://orcid.org/0000-0002-2556-3120

Contents:

Table S1. Composition of formulation Tri-C9 MNs prepared during the MNs optimization step.

Figure S1. HPLC chromatogram of dexamethasone at wavelength of 241.5 nm.

Figure S2. Tri-block C9 copolymer when dissolved in ethanol (A), chloroform (B) and cast into a PDMS mold. And (C) shows the empty mold.

Figure S3. Microscope image of MN patch made of 5% w/v Tri-C9/PVP (1:1).

Figure S4. Microscope images of MN patch containing 3% (w/v) Tri-C9 polymer (A), 5% (w/v) (B), and 10% (w/v) (C).

Figure S5. Microscope image of tri-C9 MN patch incorporated with 3% w/v PLGA in different composition (Ai) and (Bi). Height of needles after insertion into parafilm layer (Aii) and (Bii). Representative microscope images of the first layer of parafilm into which the MNs were inserted (Aiii) and (Biii).

Figure S6. Microscope image of tri-C9 MN patch incorporated with 10% w/w MCC (A). Height of needles after insertion into parafilm layer (Ai). Representative microscope image of the first layer of parafilm into which the MNs were inserted (Aii).

Figure S7. Microscope images of 3% w/v Tri-C9 MN (A), Tri-C9 MN incorporated with 5% w/w (with respect to the Tri-C9 polymer) (B) and 10% w/w (with respect to the Tri-C9 polymer) (C) PEG 3.4 kDa. (Ai, Bi, and Ci) present the needles after insertion. Representative microscope images of the first layer of parafilm into which the MNs were inserted (Aii, Bii, and Cii).

Figure S8. DSC curves for neat Tri-C9 (black), neat 35kDa PEG (green) and Tri-C9 with 10% w/w 35kDa PEG (blue).

Figure S9. SEM image of a fractured tip and yellow arrow pointing to the area analyzed by EDS (A) and the baseplate separated from the needles and analyzed individually (B).

Figure S10. Representative images of DEX-loaded MNs with PEG 35 kDa (A) and PEG 100 kDa (B) after the parafilm insertion test.

Figure S11. HPLC overlay after dissolving the gel formed at the end of the release experiment.

Table S1. Composition of formulations of Tri-C9 prepared during the MNs optimization step.

**Formulation**	**Composition**	**Tested parameter**
Tri-C9 polymer in ethanol	250 µL of 5% (w/v) Tri-C9	Effect of organic solvent
	250 µL of 5% (w/v) Tri-C9 and polyvinyl povidone (1:1)	
Tri-C9 polymer in chloroform	250 µL of 3% (w/v) Tri-C9	
Tri-C9 polymer in chloroform with sodium alginate	Tip solution -100 µL of 3%, 5% and 10%(w/v) of Tri-C9<br>Baseplate- 150 µL of 4% (w/v) Sodium alginate	Incorporation of aqueous solution as the baseplate and optimization of the concentration of Tri-C9
Tri-C9 polymer in chloroform with sodium alginate and different excipients	Tip solution -100 µL (w/v) Tri-C9 with PLGA, MCC, and PEG	Improvement of mechanical properties
	Tip solution - 50 µL (3% w/v) PLGA, 100 µL (3% w/v) Tri-C9.<br>Baseplate- 150 µL of 4% (w/v) Sodium alginate	
PEG-based MNs formulation	Tip solution -100 µL (3% w/v) Tri-C9 with 5% and 10% (w/w in regard to Tri-C9) of 3.4kDa PEG	Effect of PEG concentration and molecular weight
	Tip solution -100 µL (3% w/v) Tri-C9 with 10% (w/w in regard to Tri-C9) of 3.4kDa, 10kDa, 35kDa and 100kDa PEG<br>Baseplate- 150 µL of 4% (w/v) Sodium alginate	

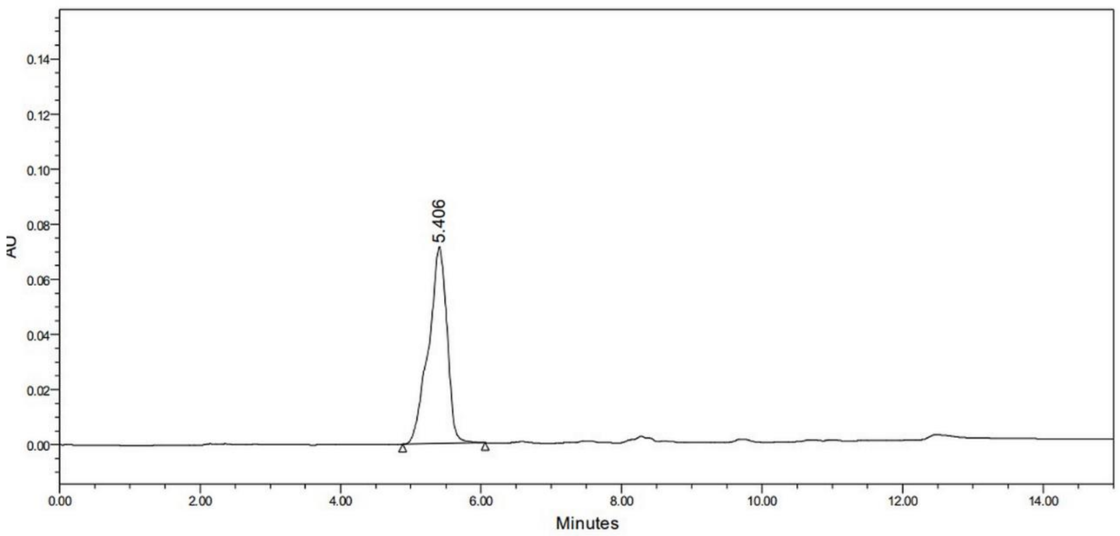

Figure S1. HPLC chromatogram of dexamethasone at wavelength of 241.5 nm.

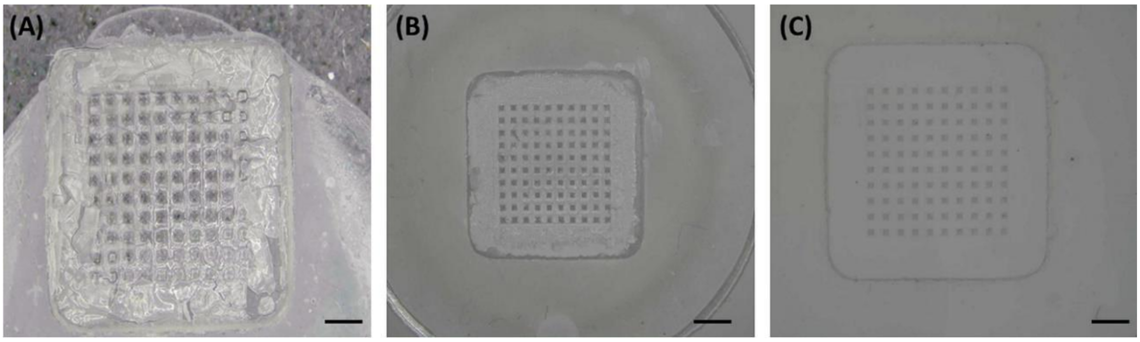

Figure S2. Tri-block C9 copolymer when dissolved in ethanol (A), chloroform (B) and cast into a PDMS mold. And (C) shows the empty mold. Scale bar 1mm.

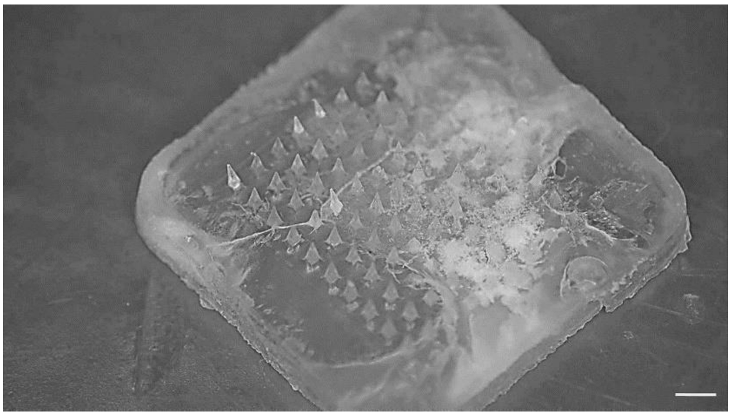

Figure S3. Microscope image represents MN patch made of 5% w/v Tri-C9/PVP (1:1). Scale bar 1mm.

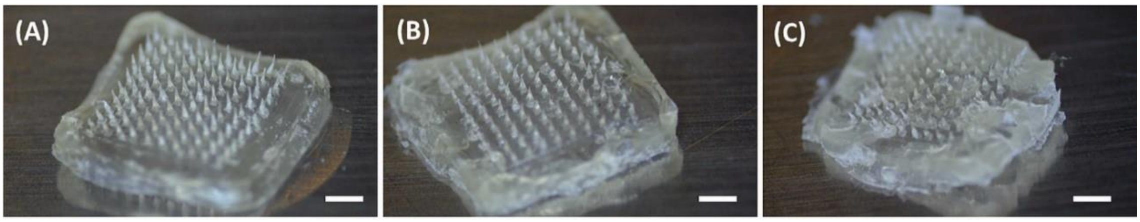

Figure S4. Microscope images of MN patch containing 3% (w/v) Tri-C9 polymer (A), 5% (w/v) (B), and 10% (w/v) (C). Scale bar 1mm.

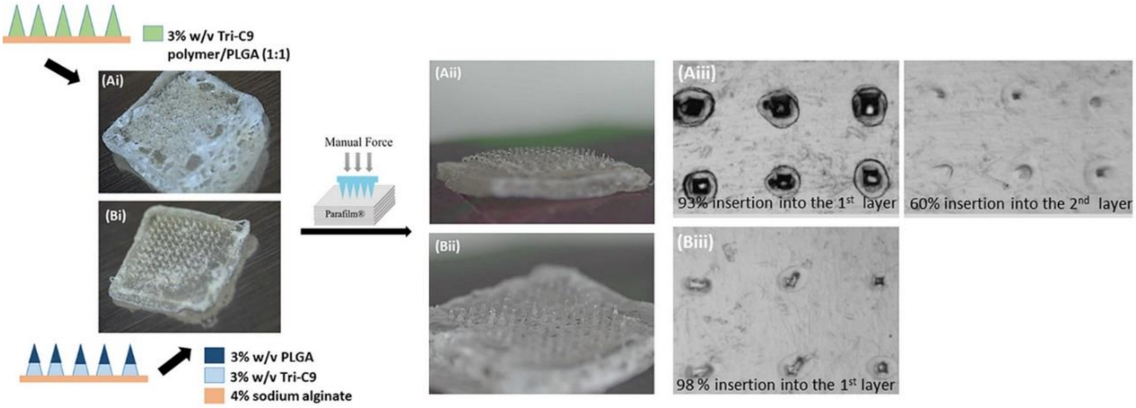

Figure S5. Microscope image of tri-C9 MN patch incorporated with 3% w/v PLGA in different composition (Ai) and (Bi). Height of needles after insertion into parafilm layer (Aii) and (Bii). Rrepresentative microscope images of the first layer of parafilm into which the MNs were inserted (Aiii) and (Biii).

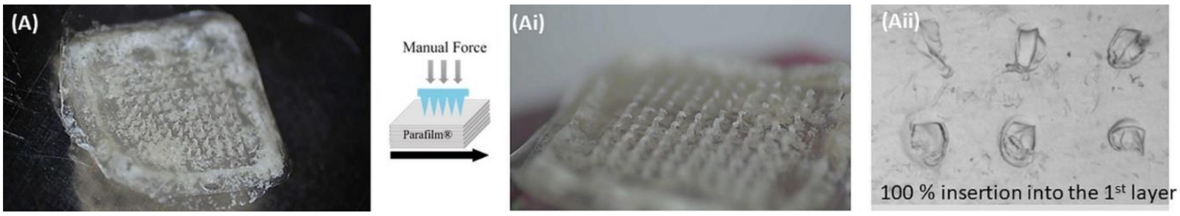

Figure S6. Microscope image of tri-C9 MN patch incorporated with 10% w/w MCC (A). Height of needles after insertion into parafilm layer (Ai). Representative microscope image of the first layer of parafilm into which the MNs were inserted (Aii).

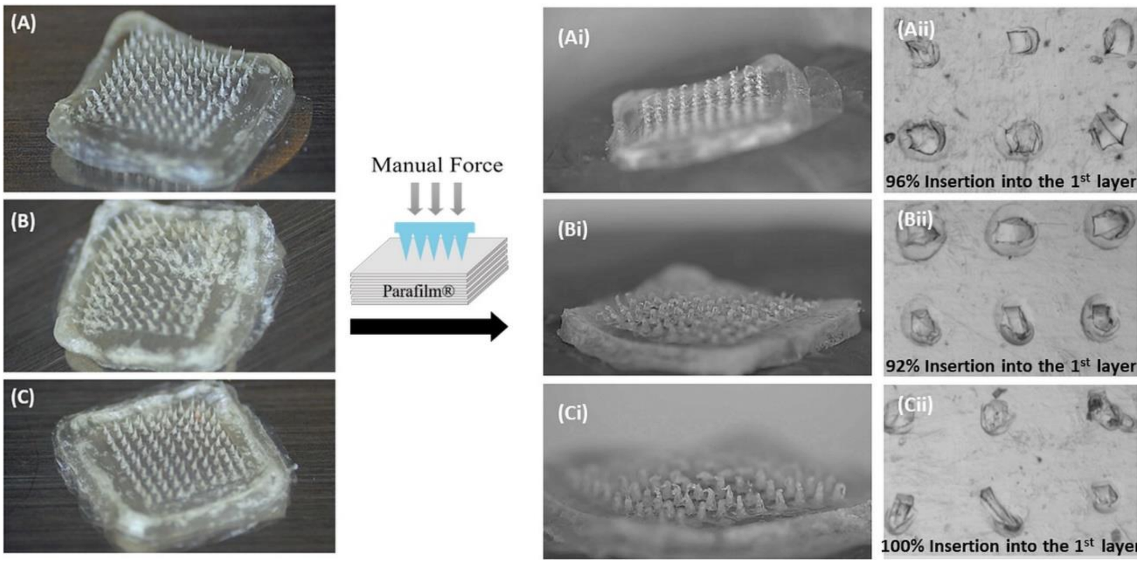

Figure S7. Microscope images of 3% w/v Tri-C9 MN (A), Tri-C9 MN incorporated with 5% w/w (with respect to the Tri-C9 polymer) (B) and 10% w/w (with respect to the Tri-C9 polymer) (C) PEG 3.4 kDa. (Ai, Bi, and Ci) present the needles after insertion. Representative microscope images of the first layer of parafilm into which the MNs were inserted (Aii, Bii, and Cii).

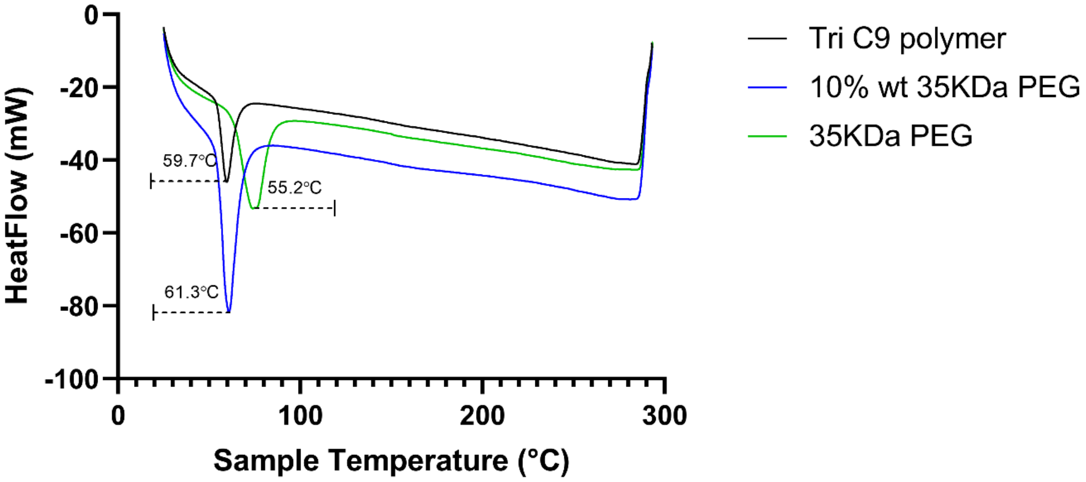

Figure S8. DSC curves for neat Tri-C9 (black), neat 35kDa PEG (green) and Tri-C9 with 10% w/w 35kDa PEG (blue).

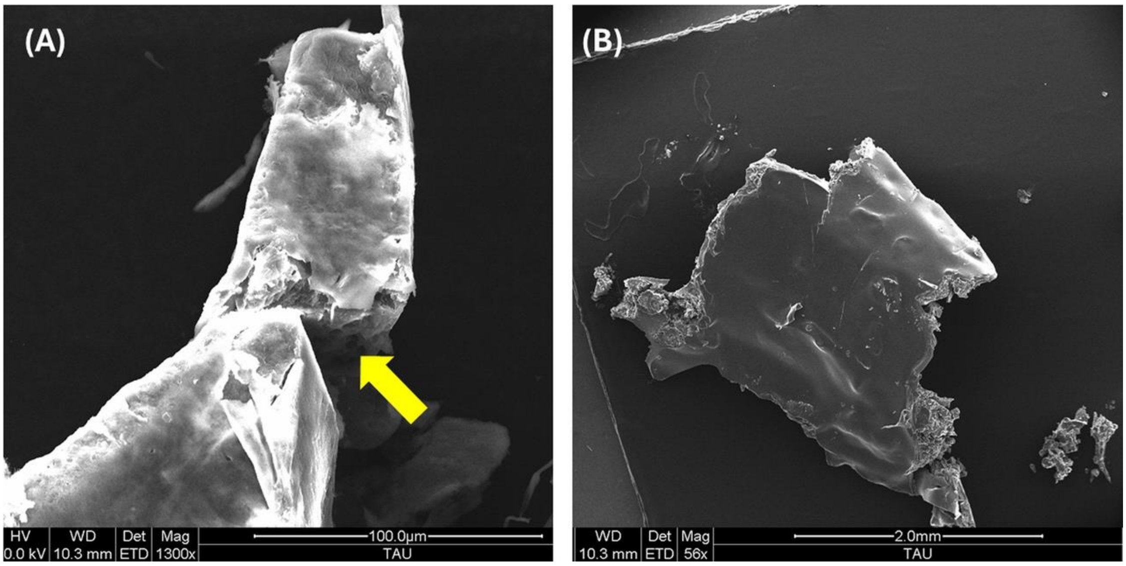

Figure S9. SEM image of a fractured tip and yellow arrow pointing to the area analyzed by EDS (A) and the baseplate separated from the needles and analyzed individually (B).

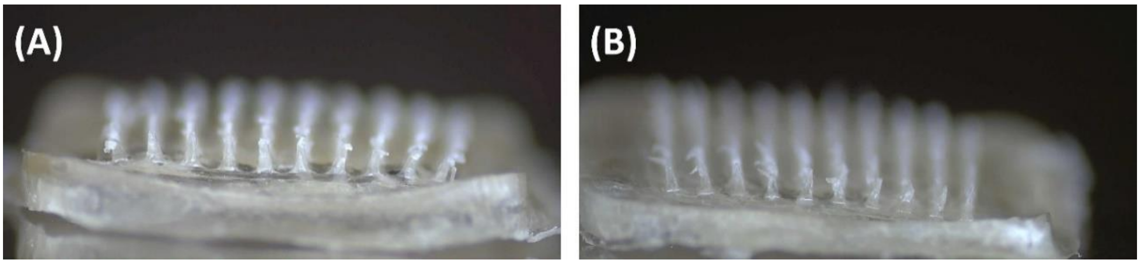

Figure S10. Representative images of DEX-loaded MNs with 35 kDa PEG (A) and 100 kDa PEG (B) after the parafilm insertion test.

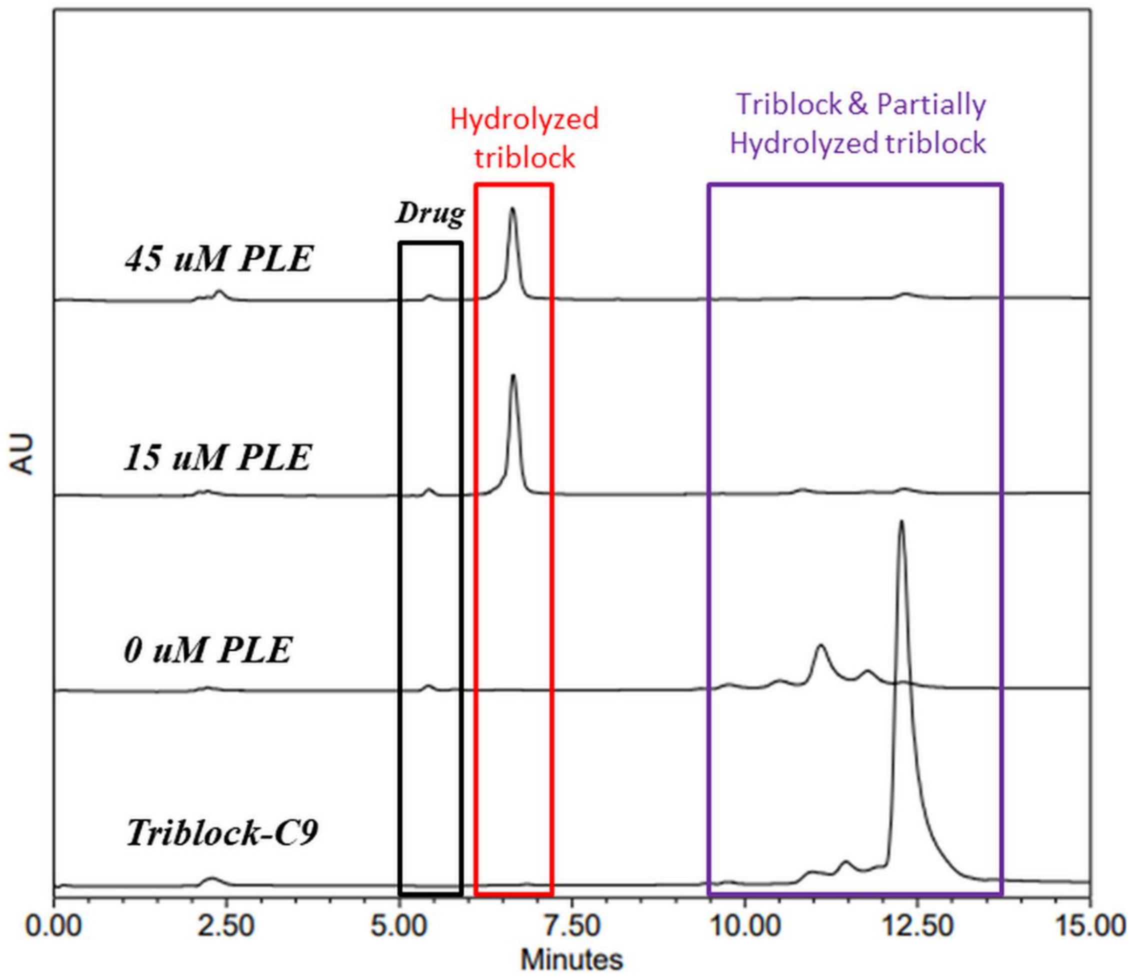

Figure S11. HPLC overlay after dissolving the gel formed at the end of the release experiment.

HPLC measurements – experimental details

Instrument: Waters Alliance e2695

Column: XBridge® Protein BEH, C4, 3.5 µm, 150x4.6 mm

Column temperature: 30°C

Sample temperature: 37°C

Solution A: 0.1% $HClO_4$: ACN 95:5v/v

Solution B: 0.1% $HClO_4$: ACN 5:95v/v

Solution C: ACN

Flow rate: 1ml/min

Gradient program for 15 minutes injection:

Time [minutes]	Sol. A [%]	Sol. B [%]	Sol. C [%]
0	75	20	5
1	75	20	5
10	0	95	5
13	0	95	5
13.1	75	20	5
15	75	20	5

Injection volume: 30 µL

Seal wash: H_2O: MeOH 90:10v/v

Needle wash: MeOH

Detector: Waters 2998 photodiode array detector

Sampling rate: 2 points/sec
